# Advertisement

**Published:** 1820

**Authors:** 


### ADVERTISEMENT.

THE *London Medical and Physical Journal* had not extended beyond the five-and-twentieth Volume, when it was found to the possessors of it to be like a Museum of Natural History, unarranged, and without a guide to direct the enquirer to the particular object of his researches. Many Subscribers, at that period, entreated the Proprietors to have an Analytical Table of its Contents constructed; it being necessary, to enable them to trace the opinions that had been advanced respecting medical science since the work was instituted, and to find the practical precepts it contained. In conformity to those entreaties, the Proprietors directed such an Index to be formed: but the extensive labour it required, deferred the completion of it until several more years had elapsed; when it became equally necessary that the Analysis should be continued up to the existing period; and a Supplement, pursuing it to the completion of the Fortieth Volume, was accordingly put in preparation, which yet caused some further delay. But, whilst they make this apology for the repeated postponement of this production, the Proprietors must express the pleasure they feel in finding it, thereby, calculated to be of increased value. It now presents an Index of reference to a mass of information on every subject of Medical Science; and on Chemistry, Botany, and Natural History, as far as they are connected with it, which, they are authorized by the assertion of the Profession to state, constitutes a complete code of theoretical and practical medicine.

Such an Index became the more necessary, from this Journal having been the medium by which much of the most important medical information advanced in England, and to a considerable extent in the United States of America, as well as on the continent of Europe, was originally conveyed to the public, and which is not to be found in other sources. This will be proved by a reference especially to the heads, Cow-Pox, Fever, Gout, Poisons, Opium, Ulcers, Venereal Diseases, Arsenic, Tetanus, Hydrocephalus, Bleeding, Uterus, Ophthalmia, Cancer, Eye, Burns, Water, Dissections, Experiments, Hydrophobia, Wounds, Mercury, Calomel, Females, Delivery, Fœtus, Parturition, Children, Consumption, Cold, Digitalis, and Puerperal Fever; and to the names of several of the most eminent modern Practitioners in various parts of the world.

The Proprietors cannot conclude this address, without expressing their grateful acknowledgments for the patronage the London Medical and Physical Journal has received from the members of the Medical Profession: their warmest hopes, with respect to the future, can only be, that it may continue to receive it in an equal degree, whilst their best exertions are made to render it worthy of the approbation of enlightened Physicians, and of important utility as a clinical guide to every practitioner of the Medical Art.

*London; December* 1, 1819.

